# ADP-Ribosylation Factor Like GTPase 4C (ARL4C) augments stem-like traits of glioblastoma cells by upregulating ALDH1A3

**DOI:** 10.7150/jca.45052

**Published:** 2021-01-01

**Authors:** Qian Chen, Wen-juan Fu, Xiao-peng Tang, Lu Wang, Qin Niu, Shuai Wang, Yong Lin, Mian-fu Cao, Rong Hu, Hai-Yan Wen, Yan Wang, Xia Zhang, Xiao-Hong Yao

**Affiliations:** 1 Institute of Pathology and Southwest Cancer Center, Southwest Hospital, Third Military Medical University (Army Medical University), and Key Laboratory of Tumor Immunopathology, Ministry of Education of China, Chongqing, China.; 2 Department of Nephrology, Southwest Hospital, Third Military Medical University (Army Medical University), Chongqing, China.; 3 Cancer Center of Daping Hospital, Third Military Medical University (Army Medical University), Chongqing, China.; 4 Department of Neurosurgery, Southwest Hospital, Third Military Medical University (Army Medical University), Chongqing, China.; 5 Department of Pathology, the Affiliated Provincial Hospital, Anhui Medical University, Hefei, China.

**Keywords:** ARL4C, glioblastoma, glioma stem-like cell, ALDH1A3

## Abstract

Glioma cells with stem cell-like properties are crucial for tumor initiation, progression and therapeutic resistance. Therefore, identifying specific factors in regulating stem-like traits is critical for the design of novel glioma therapeutics.

Herein, we reported that ADP-Ribosylation Factor Like GTPase 4C (ARL4C) was highly expressed in glioma stem-like cells (GSLCs). GSLCs, determined by the efficiency of sphere formation *in vitro* and tumor growth *in vivo*, was increased by overexpression of ARL4C. ARL4C induced the tumorigenesis through ALDH1A3. Analyses of 325 patient specimens showed that ARL4C was highly expressed in glioblastoma (GBM) as compared with lower grade gliomas. In addition, higher level ARL4C expression in glioma was correlated with poorer progression-free survival and overall survival of patients. Therefore, ARL4C may act as a novel prognostic marker and a therapeutic target for GBM.

## Introduction

Glioblastoma (GBM) is featured with heterogeneity, increased invasiveness, high recurrence rate and resistance to therapy, which were attributed to the presence of glioma stem-like cells (GSLCs) in tumors 1, 2 Therefore, it is important to identify novel approaches targeting GSLCs.

Small GTPases of Ras superfamily are composed of more than 100 members, which share a conserved structure and biochemical properties, acting as binary molecular switches turned on by binding GTP and off by hydrolyzing GTP to GDP 3. The key function of Ras superfamily members is the regulation of actin cytoskeleton rearrangement, cell growth and sustaining cell stemness 4,5. Small monomeric GTPases of Ras superfamily also include the most prominent druggable cancer targets 6, such as CDC42, RAC1 and RhoA, which are important for the growth of GBM cells 7. However, the precise role of Ras superfamily proteins in affecting GSLC features remains unclear.

ARL4C, a member of Ras superfamily, is associated with migration, invasion and proliferation of cancer cells and is regarded as a therapeutic target in colorectal cancer, lung cancer 8 and liver cancer 9. We previously reported that high level expression of ARL4C promoted the invasion of PTEN-deficient GBM cells 10. In order to examine the clinical significance of ARL4C and its role in controlling GSLCs, we evaluated the contribution of ARL4C to sustaining the stemness of GSLCs and the underlying molecular mechanism. In addition, we analyzed the expression of ARL4C in clinical glioma specimens and its association with patient prognosis.

## Materials and Methods

### Cell culture

The human GBM cell lines U87-MG, T98G from the ATCC were cultured as described 10. Human normal glial cell line HEB was generously provided by Professor Guang-mei Yan (Department of Pharmacology, Sun Yat-sen University, Guangzhou, China). Primary GBM cells (GBM-1) were obtained from GBM tumor tissues (Southwest Hospital, AMU, China) and cultured as previously described 11.

GSLCs derived from GBM cells (U87-MG and GBM-1) were cultured with Neurobasal medium (Life Technologies, Carlsbad, CA, USA) with EGF (20 ng/ml, PeproTech, Rocky Hill, NJ, USA), bFGF (20 ng/ml, PeproTech) and B27 Supplement (20 μl/ml, Life Technologies, Carlsbad, CA, USA).

### Patients and tissue specimens

This study included three independent cohorts of glioma patients, who did not receive preoperative radiotherapy, chemotherapy or immunotherapy. In cohort of Chongqing, glioma tissue samples were collected during the years 2013 to 2015. In cohort of Hefei, samples were collected during the years 2007 to 2011. In the Shanghai cohort, glioma tissue microarrays were purchased from BioChip (Shanghai, China), which were collected during the years from 2008 to 2011. All patients were followed up for 5 years and clinic pathologic parameters were obtained from medical records maintained in the Departments of Pathology of respective hospitals (Supplementary Table 1). The study concerning human specimens were performed in accordance with the principles of the Helsinki Declaration and approved by the Ethics Committee of Third Military Medical University (Army Medical University), Chongqing, China and the Affiliated Provincial Hospital of Anhui Medical University, Hefei, China. Glioma diagnosis was based on the World Health Organization (WHO) Classification of central nervous system tumor (2016).

### Immunohistochemical staining

Immunohistochemical staining was conducted as previously described 12. Paraffin-embedded GBM tissues and xenografts were cut into 5 μm-thick sections using a Dako REAL Envision Detection System (Dako, Glostrup, Denmark) according to manufacturer's instructions. The slides were incubated with rabbit polyclonal anti-ARL4C (1:100; ab-122025, Abcam, Cambridge, UK) at 4°C overnight. After removing the primary antibody with PBS, a horseradish per-oxidase (HRP)-conjugated secondary antibody (Dako) was added and the slides were incubated at 37°C for 30 min. Semi-quantitation of ARL4C was independently performed according to the staining intensity and the percentage of positive tumor cells as previously described 13. The staining intensity was scored as follows: 0, no staining; 1, weak staining; 2, moderate staining; and 3, strong staining. The percentage of positive cancer cells was scored as: 1 = 1-25%; 2 = 26-50%; 3 = 51-75%; and 4 > 75%. All slides were evaluated independently by two pathologists without knowledge of the identity of patients and the clinical outcome.

### Western blotting and qPCR

Western blotting was conducted as previously described 12. Primary antibodies used were: anti-ARL4C (1:500, ab-122025, Abcam), anti-ALDH1A3 (1:500, ab-129815, Abcam), anti-OCT4 (1:1000, CST, Danvers, MA, USA) and anti-SOX2 (1:1000, CST, Danvers, MA, USA).

Total RNA was extracted from the cell lines with RNAiso reagent according to the manufacturer's protocol. Reverse-transcription and quantitative Real-time PCR were performed using a One Step SYBRPrimer: ARL4C F: 5-CCAGTCCCTGCATATCGT-CAT-3; R: 3-TTCACGAACTCGTTGAACTTGA-5; RAB26; F: 5-GTCTGCTGGTGC GATTCAAG-3; R: 3-GCATGGGTAACACTGC GGA-5; RAB27B F: 5-AACAAGGC AGACCTACCA GAT-3; R: 3-TTCCCCATCCAAGTTTCCAGA-5'; RAB6B: F: 5-TGTACGACAGCTTCGACAACA-3; R: 3-CTGCGGAACCTCTCCTGAC-5.

### *In vitro* limiting dilution and sphere forming assays

*In vitro* limiting dilution assay was performed as previously described 13 and analyzed with an online software (http://bioinf.wehi.edu.au/software/elda/). The sphere with a diameter > 50 μm was counted under microscopy.

### Lentiviral infection procedures

For overexpression of ARL4C in GBM cells, full length human ARL4C were generated and inserted into a lentivirus vector. Lentiviral particles containing ARL4C were packaged. GBM-1 and U87 cells (1×10^6^/well) were infected with lentivirus containing constructs. The cells were selected and enriched by 4 µg/ml puromycin.

GBM-1 and U87 cells (1×10^6^/well) were infected with sh*ARL4C* lentivirus or empty control lentivirus vectors (Genechem, Shanghai, China). Cells were selected and enriched by 4 μg/ml puromycin. The sequence for the shRNAs were listed in Supplementary Table 2.

### cDNA microarray analysis

Affymetrix Whole Human Genome Oligo Microarray analysis was conducted by Shanghai Genechem Corporation (Shanghai, China). Genes differentially expressed with logarithmic ratios exceeding 2-folds were defined significantly (*p* < 0.05). The gene chip data on shARL4C-1 and shCtrl-GBM-1 were deposited under accession number GSE121253 in NCBI Gene Expression Omnibus (GEO).

### Tumor implantation

Five-week-old female NOD-SCID mice were purchased from Laboratory Animal Center of Southwest Hospital, Third Military Medical University (Army Medical University) (Chongqing, China). pLVX-eGFP-linker-luciferase lentivirus transfected U87-MG cells (1×10^5^ /μl) were injected intracranially into the right frontal lobes of the mice. Two or four weeks later, xenograft tumors were quantified by bioluminescence imaging using an *In vivo* Image System (IVIS) Spectrum. Tumor-bearing mice were sacrificed to collect tumors when the animals became moribund. Animal experiments were approved by the Institutional Animal Care and Use Committee of Southwest Hospital, Third Military Medical University (Army Medical University) in accordance with the Guide for the Care and Use of Laboratory Animals.

### Statistical analysis

All experiments were performed at least three times and data were shown as the mean ± SD. Unpaired two-group comparison and multiple comparisons were made with the Student t-test or one-way ANOVA. Animal survival was analyzed using the Kaplan-Meier method, with the log-rank test for comparison. X-tile software was used to determine the cutoff point of ARL4C expression in clinical sample analysis 14.

## Results

### ARL4C is preferentially expressed in glioma stem-like cells

**Figure 1:**
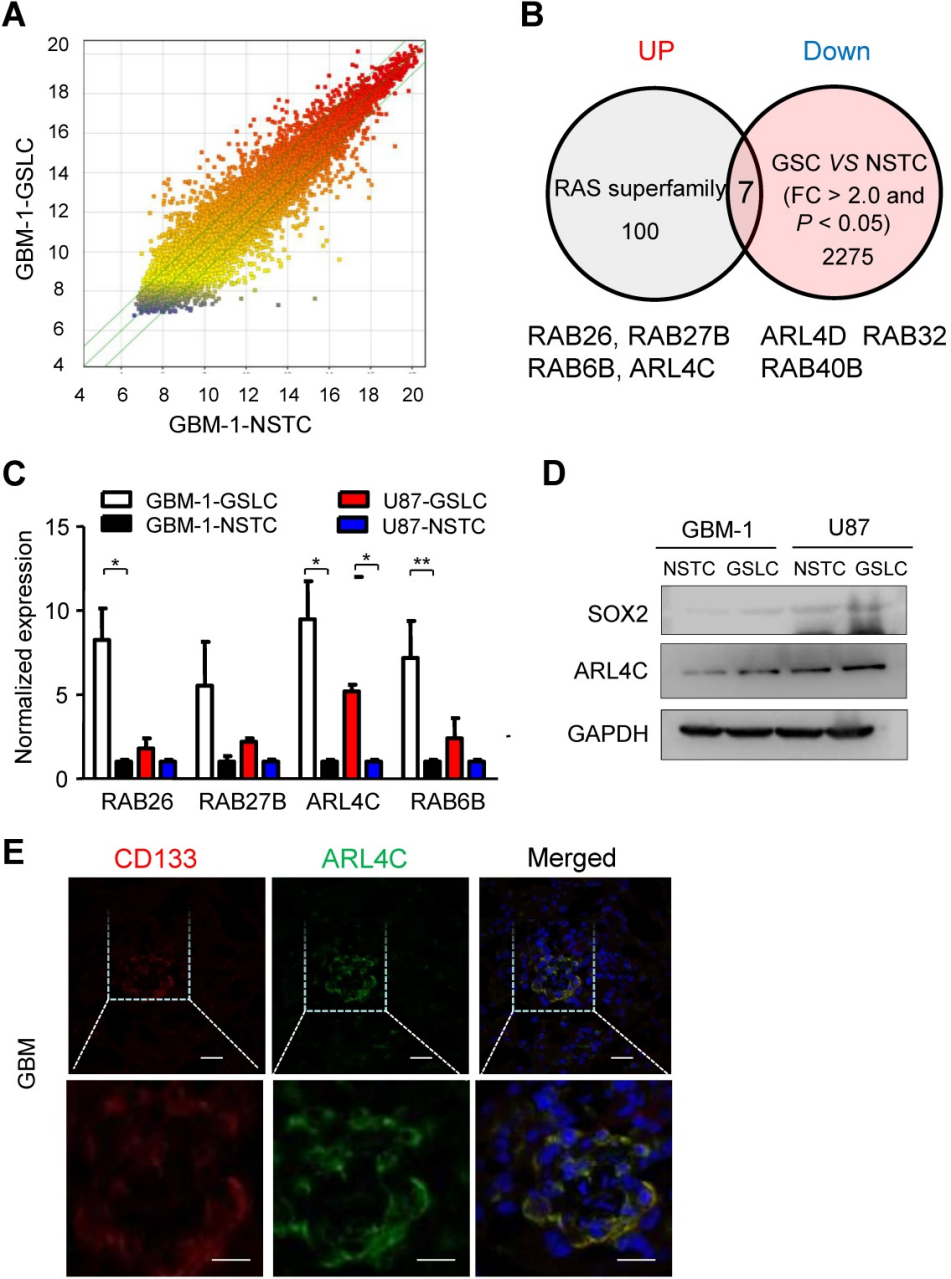
** ARL4C is preferentially expressed in GSLCs**. (**A**) Scattered map of gene expression in GSLCs relative to NSTCs from genechip. A total of 2275 genes were identified with > 2.0 fold changes. (**B**) Seven genes were screened by comparison of Ras superfamily sets. Four candidates, RAB26, RAB27B, RAB6B, and ARL4C were significantly upregulated in GSLCs. (**C**) QRT-PCR analyses showing the expressions of RAB26, RAB27B, RAB6B, and ARL4D in GSLCs (n = 2) relative to matched NSTCs (n = 2). (**D**) Western blotting indicating the preferential expression of ARL4C and the GSLC marker SOX2 in GSLCs (n = 6) as compared with NSTCs. (**e**) ARL4C colocalized with CD133 in frozen GBM tissues. Scale bar = 20 µm; CD133: red; ARL4C: green; nucleus: blue.

As Ras-superfamily members have been linked with tumor initiation and progression, we aimed to identify specific small GTPases of the superfamily that may be expressed in glioma stem-like cells (GSLCs) and examine their functional significance in tumor formation and progression. Microarray profiles comparing GSLCs and non-stem tumor cells (NSTCs) isolated from primary human GBM (GBM-1) (Fig. 1A) showed that 4 out of 100 members in the Ras-superfamily (RAB26, RAB27B, RAB6B and ARL4C) were significantly up-regulated in GSLCs (*P* < 0.05) (Fig. 1B). QRT-PCR indicated that ARL4C was consistently increased in all tested GSLCs compared with matched NSTCs (Fig. 1C). Increased expression of ARL4C protein in GSLCs relative to NSTCs was then confirmed (Fig. 1D) and co-localized with the GSLC marker CD133 in human GBM tissues (Fig. 1E).

### ARL4C is required for the self-renewal and proliferation of GBM cell *in vitro* and *in vivo*

**Figure 2:**
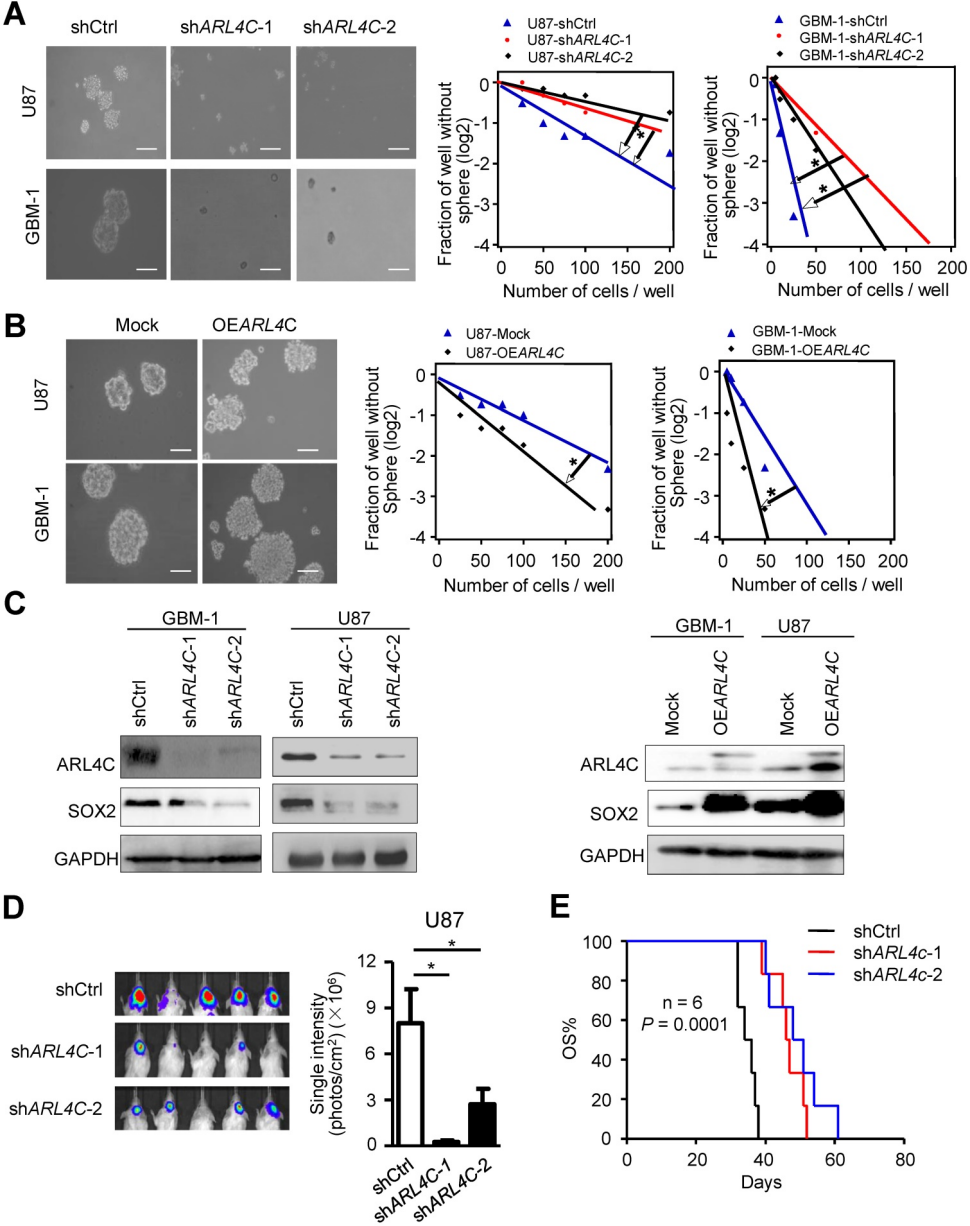
** ARL4C increases the stem-like traits of GBM cells *in vitro* and their tumorigenesis* in vivo***. (**A**) Representative images (*Left panel*) and sphere-forming efficiency (*Right panel*) of sh*ARL4C*-1, sh*ARL4C*-2 and shCtrl GBM cells. Scale bar = 100 µm. (**B**) Representative images (*Left panel*) and sphere-forming efficiency (*Right panel*) of OE*ARL4C* GBM cells and Mock cells. Scale bar = 100 µm. (**C-D**) Western blotting of SOX2 in cell groups with down- (**C**) and upregulated -(**D**) ARL4C as compared with control cells. (**E**) Bioluminescence images (*Left panel*) and quantification (*Right panel*) of tumors in NOD-SCID mice implanted with shCtrl, sh*ARL4C-1*-and sh*ARL4C-*2 U87 cells. (**F**) Survival curves of tumor-bearing mice implanted with shCtrl, sh*ARL4C*1 and sh*ARL4*C2 U87 cells (*P < 0.05; **P < 0.01).

We found ARL4C was highly expressed in GBM cells as compared with normal brain glial cells (Supplementary Fig. 1A). We then overexpressed or knocked down ARL4C in GBM cells and found decreased sphere-forming efficiency of sh*ARL4C* GBM cells as compared with shCtrl cells (Fig. 2A). In contrast, overexpressing ARL4C in GBM cells increased their sphere-forming capabilities (Fig. 2B and Supplementary Fig. 1B, 1C). Fig. 2C showed that expression of SOX2 decreased in sh*ARL4C* GBM cells. However, overexpression of ARL4C increased SOX2 in tumor cells.

To further examine the effects of ARL4C on tumorigenesis of GSLCs *in vivo*, we transplanted sh*ARL4C* and shCtrl GBM cells into the brain of NOD-SCID mice. Bioluminescent imaging detected tumors formed by sh*ARL4C* U87 cells with smaller volumes compared to those formed by shCtrl U87 cells (Fig. 2D). Mice bearing tumors formed by sh*ARL4C* U87 cells showed longer survival than those transplanted with shCtrl U87 cells (Fig.2E). These data suggested that ARL4C was crucial for the tumorigenesis of GBM cells.

### The ARL4C/ALDH1A3 axis facilitated the tumorigenesis of GBM cells

**Figure 3:**
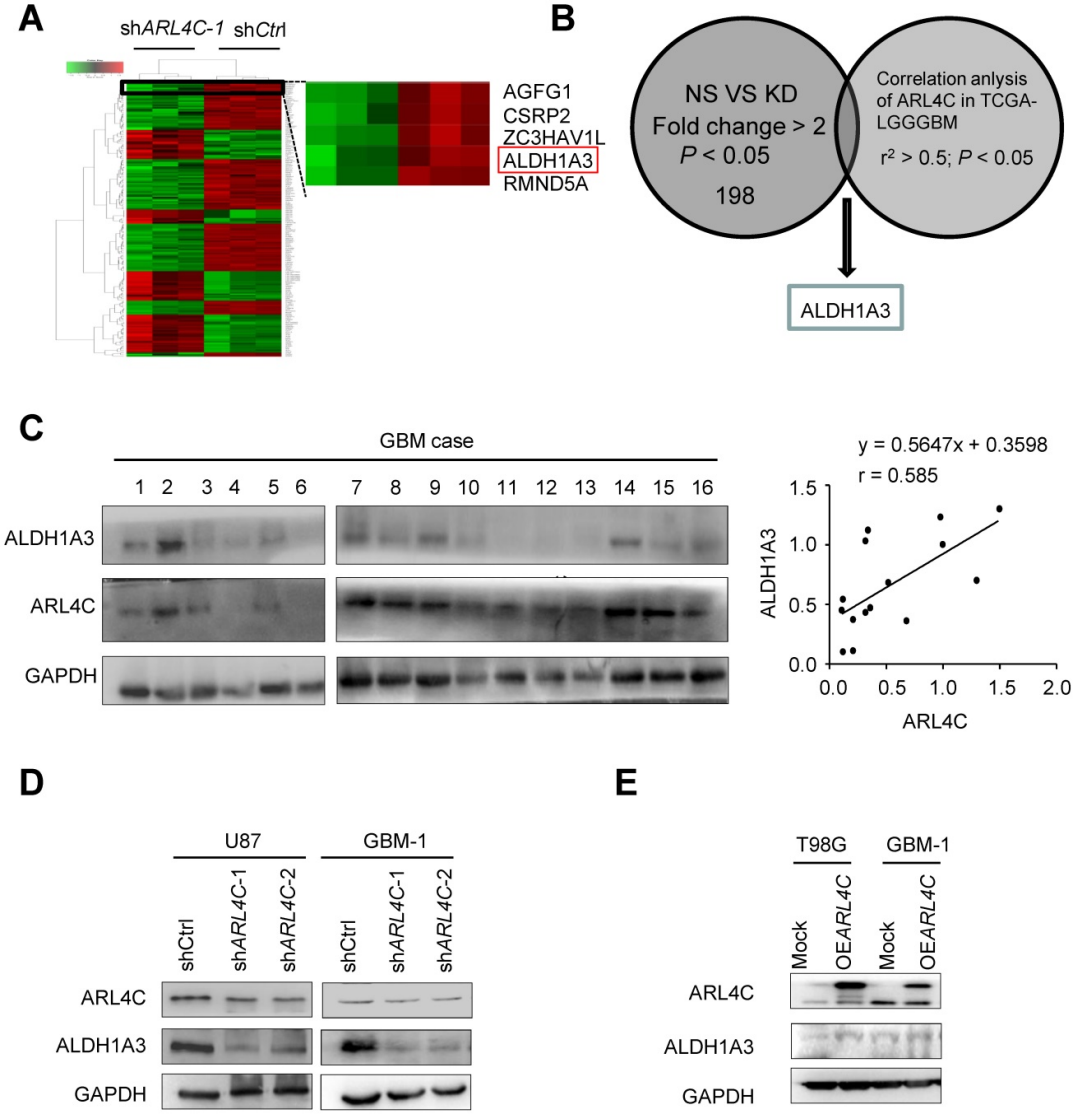
** ARL4C augments the tumorigenesis of glioma cells by upregulating ALDH1A3 expression**. (**A**) Heatmap depicting 198 transcripts differentially expressed in shCtrl and sh*ARL4C*1 GBM cells as indicated by > 2.0 fold changes. (**B**) ALDH1A3 gene identified by interacting with differential gene sets and correlation analysis of ARL4C expression in TCGA-LGGGBM database as indicated by r^2^> 0.5. (**C**) Western blotting (*Right panel*) and statistical analysis (*Left panel*) of ARL4C and ALDH1A3 expression in 16 human GBM tissues. (**D**) Western blotting of ALDH1A3 in sh*ARL4C*-1-, sh*ARL4C*-2-, and shCtrl GBM cells. (**E**) Western blotting of ALDH1A3 in Mock and OE*ARL4C* GBM cells.

**Figure 4:**
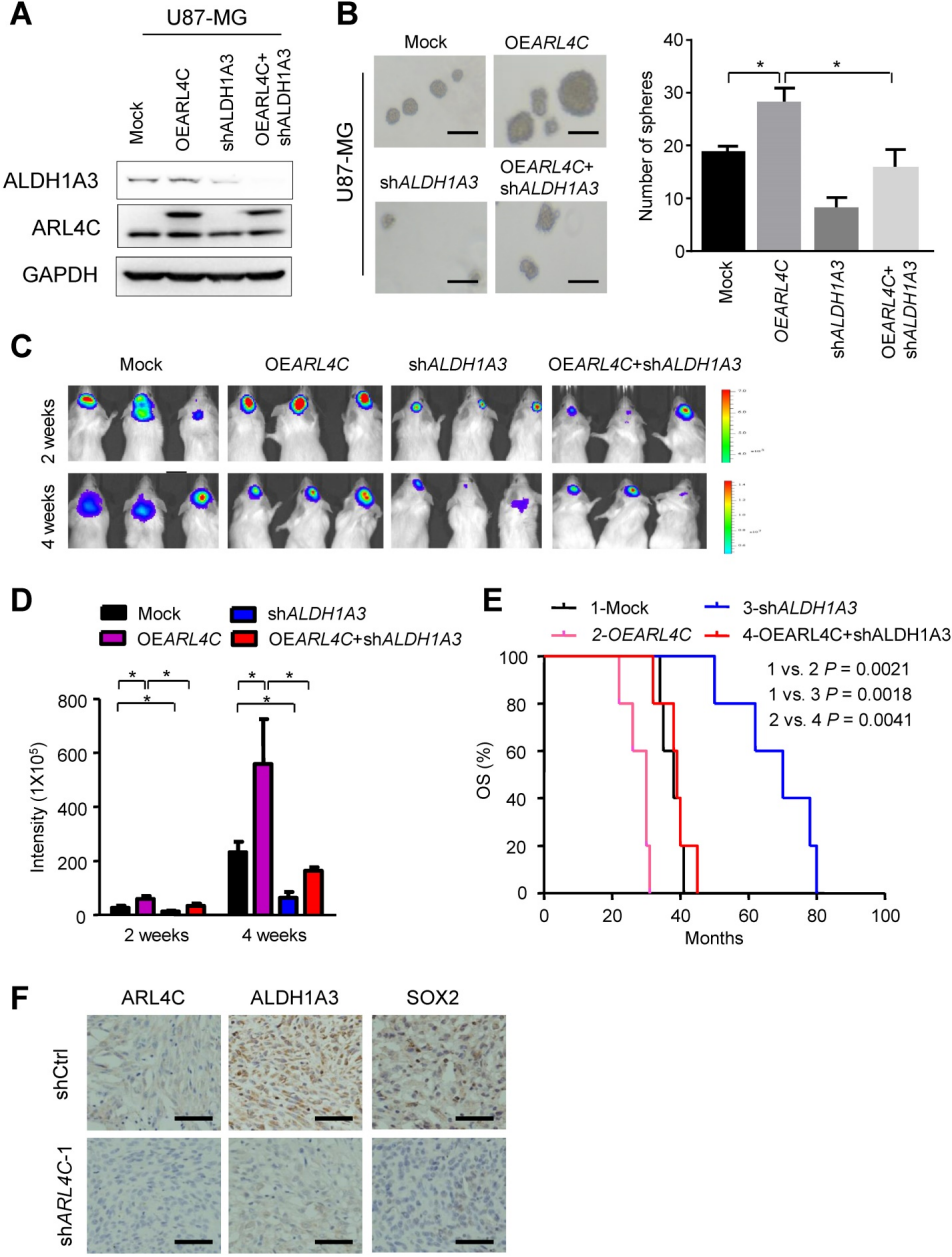
** ARL4C/ALDH1A3 promotes the progression of GBM xenografts *in vitro and vivo*. (A)** Immunoblotting of ALDH1A3 and ARL4C in U87 cells overexpressing ARL4C (OE*ARL4C*), ALDH1A3 knocking down (sh*ALDH1A3*), OE*ARL4C* + sh*ALDH1A3* and control vectors (Mock) cells. (**B**) Representative images (*Left panel*) and sphere-forming efficiency (*Right panel*) of mock, OE*ARL4C* and sh*ALDH1A3*, OE*ARL4C* + sh*ALDH1A3* GBM cells. Scale bar = 100 µm. (**C-D**) Representative bioluminescence images (**C**) and quantification (**D**) of 2- and 4- weeks xenograft tumors formed by U87 cells overexpressing ARL4C (OE*ARL4C*), ALDH1A3 knocking down (sh*ALDH1A3*), OE*ARL4C* + sh*ALDH1A3* and control vectors (Mock) cells. (**E**) Kaplan-Meier survival analysis of mice bearing tumors formed by sh*ARL4C*, sh*ALDH1A3*, OE*ARL4C* + sh*ALDH1A3* and Mock U87 cells. *P < 0.05; **P < 0.01. (**F**) Representative IHC staining for ARL4C, ALDH1A3 and SOX2 in tumors formed by shCtrl and sh*ARL4C*-1 GBM cells. Scale bar = 50 µm.

Microarray analyses were then performed to compare shCtrl-GBM-1 with sh*ARL4C*-1-GBM-1 cells for gene expression (Fig. 3A), ALDH1A3, a predominant ALDH enzyme that participates in diverse range of biological processes in glioma stem cells 15,16, was most correlated to ARL4C expression in TCGA-LGGGBM database among 198 genes selected by comparison of differential gene expression in shCtrl- and sh*ARL4C-*1 GBM-1 cells (Fig. 3B). Western blotting showed that the expression of ALDH1A3 was higher in ARL4C-high GBM specimens than in ARL4C-low GBM specimens (Fig. 3C). Additionally, ALDH1A3 was decreased in sh*ARL4C*-1 GBM cells than that in shCtrl cells, and with an increase in OE*ARL*4C GBM cells (Fig. 3D and 3E). When overexpressing ARL4C or knocking down of ALDH1A3 in U87 cells (Fig. 4A and 4B), we found that ARL4C increased the sphere forming ability of GBM cell, which is partially decreased by the knockdown of ALDH1A3. In examining the capacity of ALDH1A3 knockdown to reverse tumor growth, we found that knockdown of ALDH1A3 attenuated the pro-oncogene effects of ARL4C overexpressed in GBM cells (Fig. 4C and 4D). The survival of mice bearing-tumor formed by sh*ALDH1A3* and OE*ARL4C*-cells were markedly increased as compared to those bearing tumors formed by OE*ARL4C*-cells alone (Fig. 4E). IHC staining of xenograft tumors showed that the expression of ALDH1A3 and SOX2 was decreased in tumors derived from sh*ARL4C*-1-GBM cells as compared with those derived from shCtrl-GBM cells (Fig. 4F). Thus, ALDH1A3 appears to be a target in ARL4C-mediated tumorigenic process.

### ARL4C was associated to poor prognosis in patients with high grade glioma

**Figure 5:**
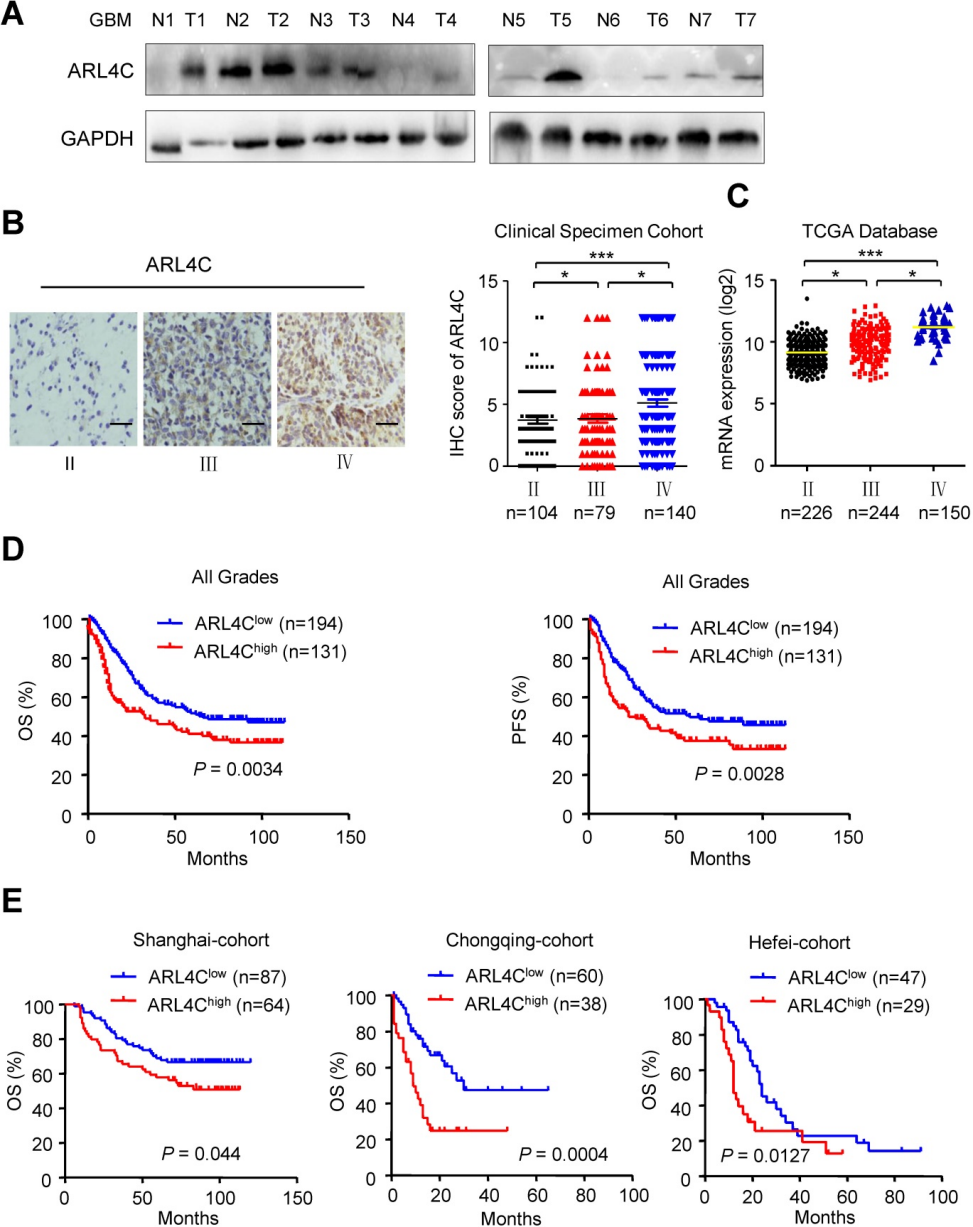
** ARL4C is highly expressed in human gliomas and correlated with poor prognosis**. (**A**) Western blotting showing the expression of ARL4C in seven pairs of glioma tumor tissues (T) and corresponding adjacent normal tissues (N). (**B**) Representative images (*Left panel*) and IHC score (*Right panel*) of ARL4C staining in tumors from patients with different grade gliomas. Scare bar = 50 µm. (**C**) ARL4C expression in tumors with different grades from TCGA-database. (**D**) Overall survival and progression free survival rate of patients in low *versus* high ARL4C expressing gliomas. (**E**) Kaplan-Meier curves of the overall survival rate of glioma patients with high *versus* low ARL4C expressing tumors in Shanghai, Chongqing and HeFei.

Validating the relationship between ARL4C expression and human glioma malignancy revealed that human gliomas expressed relatively higher levels of ARL4C than corresponding normal brain tissues (Fig. 5A). In 325 patients with gliomas, ARL4C was markedly increased in high-grade tumors. Furthermore, ARL4C expression in Grade IV GBM was higher than that in Grade III anaplastic gliomas (*P* < 0.05; Fig. 5B). Similar results were obtained from 620 patients with gliomas from TCGA database (Fig. 5C).

Kaplan-Meier analyses showed that patients with lower level of ARL4C had longer overall survival (OS; *P* = 0.0043) and progression-free survival (PFS;* P* = 0.0028) than those with higher level ARL4C in tumors. Similar results were obtained in all three regional-cohorts. Additionally, higher ARL4C expression was significantly correlated with poorer progression-free survival and overall survival of patients with Grade IV GBM (Supplementary Fig. 2A). In contrast, no correlation between ARL4C and patient survival was observed in patients with Grade Π and Grade III tumors (Supplementary Fig. 2B and 2C). Therefore, higher ARL4C expression was associated with poorer prognosis in gliomas and ARL4C serves as a predictor.

## Discussion

Glioma stem like cells (GSLCs), a subset of tumor cells with stem cell characteristics such as the preferential expression of stem cell markers and the enhanced self-renewal ability, are crucial for tumorigenesis, progression and control of the plasticity of tumor cells 17,18.Given the distinct gene sets and signaling pathways differentially expressed in gliomas with different grades, GSLCs in each grade may contain diverse and dysregulated pathways that govern unique phenotype of tumor growth, progression, and resistance to therapy 19,20. Our current studies found that ARL4C was highly expressed in GSLCs and contributed to their stemness. We also showed that ARL4C was associated with tumor grade and poorer OS of patients, suggesting that ARL4C acts as an oncoprotein in glioma.

ARL4C, a Ras-GTP binding protein, was involved in various tumor progression processes 21,22. The expression of ARL4C is regulated by many factors and pathways, such as promoter methylation, Wnt-β-catenin and growth factor-Ras signaling 23,24. Our previous study found that PTEN/AKT/mTOR pathway stabilized ARL4C and decreased its ubiquitination. However, the downstream pathway of ARL4C was unknown. Here, we identified that ALDH1A3 as the main downstream target of ARL4C, and that ARL4C/ALDH1A3 axis was crucial for the tumorigenicity of glioma cells.

ALDH1A3 is a member of ALDH superfamily, which is vital for oxidizing endogenous and exogenous aldehydes 25. Elevated ALDH has been considered a cancer stem cell marker in multiple tumor types 26. Recent studies have shown that ALDH1A3 was required for sustaining ALDH activity in cancer stem cells 27 and was also regarded as a key determinant for the maintenance of mesochymal features of GSLCs. ALDH1A3 expression is regulated by several factors including promoter methylation, STAT3, hepatocyte growth factor (HGF)/c-MET pathways and protein ubiquitination. Our study defined ARL4C as a novel regulator that increases ALDH1A3 activity in GSLCs.

In summary, we provided evidence for ARL4C-ALDH1A3 axis in regulating GSLC properties and its value as a predictor of glioma patient survival.

## Supplementary Material

Supplementary figures and tables.
