# Supplementary material for: ADP-Ribosylation Factor Like GTPase 4C (ARL4C) augments stem-like traits of glioblastoma cells by upregulating ALDH1A3
Source: J Cancer. 2021 Jan 1;12(3):818–26. doi: 10.7150/jca.45052 (PMC7778538; doi:10.7150/jca.45052)
Supplement: Supplementary file 1 — Supplementary figures and tables. [file jcav12p0818s1.pdf]

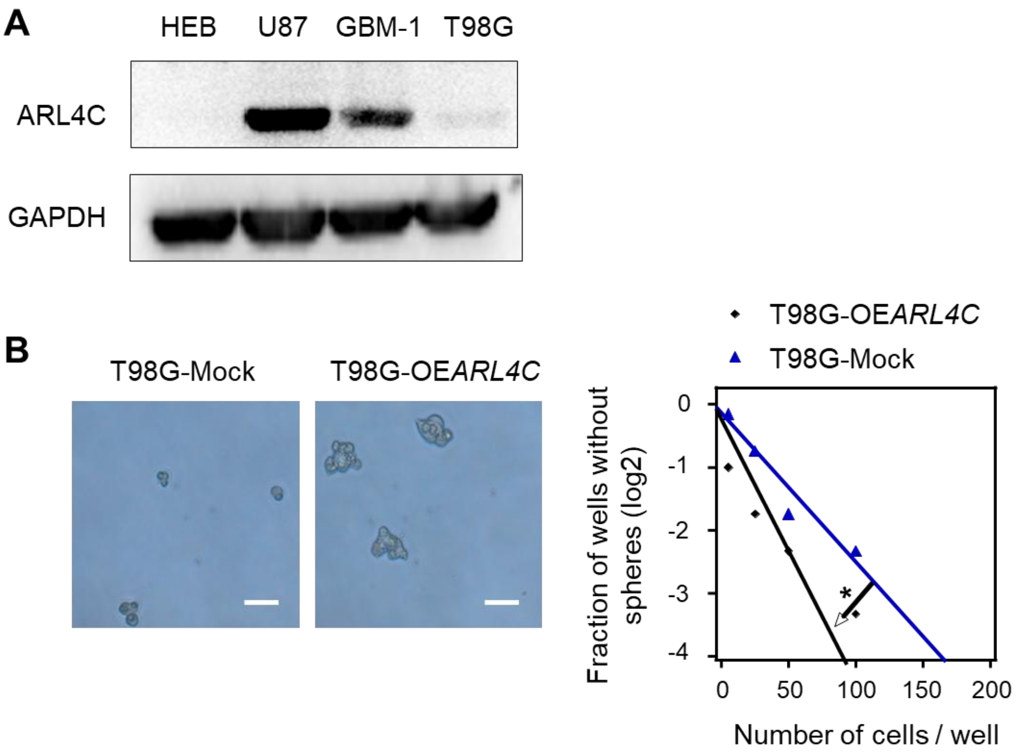

Supplementary Figure 1. High expression of ARL4C in GBM cell is correlated with the stemness. (A) Expression of ARL4C in human normal glial cell line (HEB) and GBM cells (GBM-1, U87 and T98G). (B) Representative images (*Left panel*) and sphere-forming efficiency (*Right panel*) of Mock and OE*ARL4C* T98G cells . Scale bar = 100 μm.

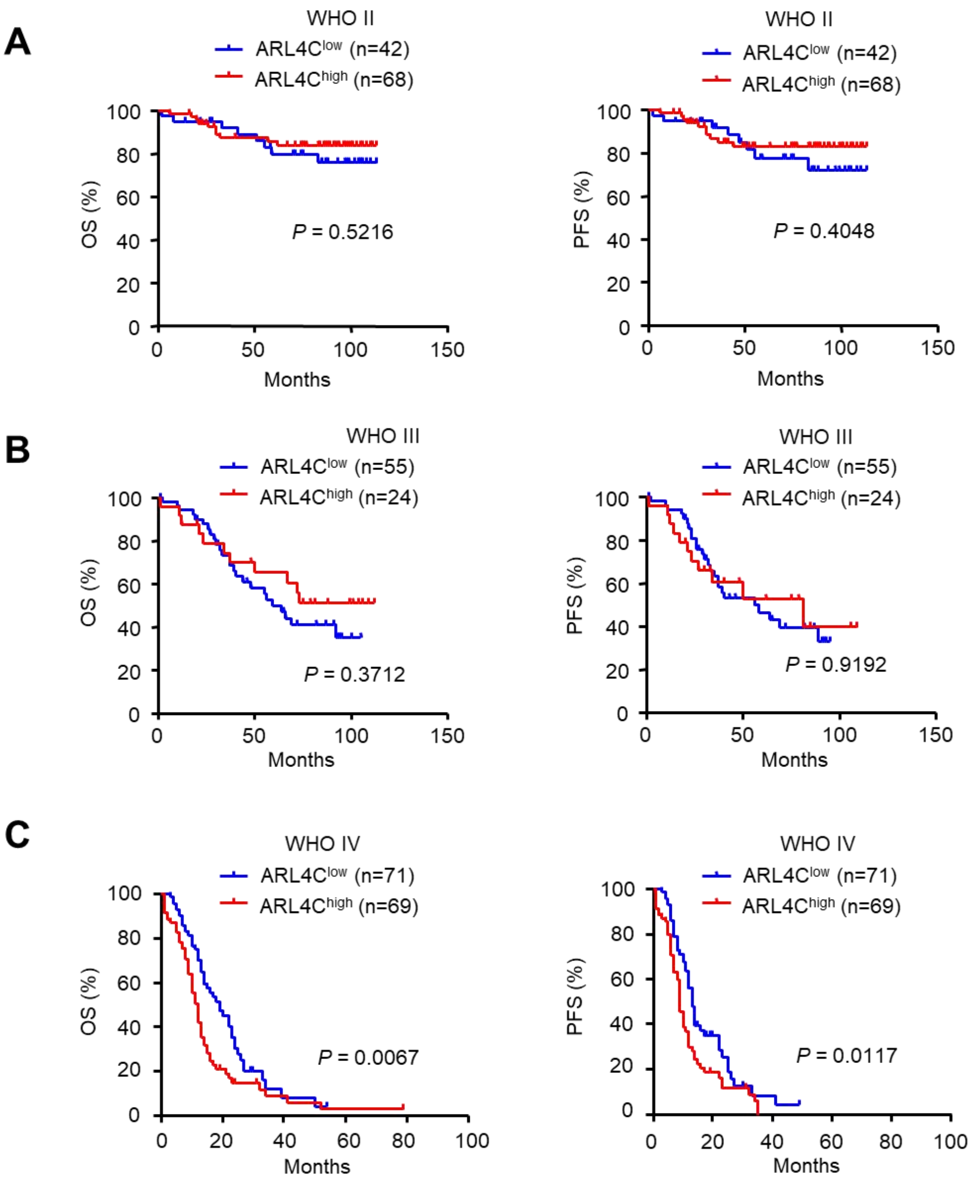

Supplementary Figure 2. High level expression of ARL4C is correlated with poor prognosis of GBM patient. (A-B) Overall survival rate (OS) and progression free survival rate (PFS) of patients with ARL4C-low versus ARL4C-high glioma (WHO II and III). (C) OS and PFS rate of patients with ARL4C-low versus ARL4C-high GBM.

Supplementary Table 1. The clinical features of the glioma specimens used in this study.

Feature	Number (Percentage)
Gender	
Male	193 (59.39%)
Female	132 (40.61%)
Age (year, median ± SD)	44.74 ± 16.80
Predominant side of tumor location	
Left	146 (44.92%)
Right	143 (44.00%)
Middle	36 (11.08%)
Predominant lobe of tumor location	
Frontal	116 (35.69%)
Temporal	101 (31.07%)
Parietal	33 (10.15%)
others	75 (23.09%)
Surgical resection	
GTR	283 (87.07%)
PR	42 (12.93%)
Grade	
II	106 (32.62%)
III	79 (24.31%)
IV	140 (43.08%)
Region	
Chongqing	98 (30.1%)
Hefei	76 (23.4%)
Shanghai	151 (46.5%)

Abbreviation: SD, Stardard deviation; GTR, Gross total resection; PR, Partial resection.

Supplementary Table 2. The sequences of shRNA.

**shRNA / siRNA**	**Sequence (5′ - 3′)**
sh*ARL4C*1	TGATCCTGAAACGCAGGAA
sh*ARL4C*2	TCAAGTTCAACGAGTTCGT
shCtrl	TTCTCCGAACGTGTCACGT
